# Supplementary material for: Risk factors for sacrococcygeal pilonidal sinus: a systematic review and meta-analysis supplemented by genetic causal assessment
Source: Front Surg. 2026 Jan 7;12:1718589. doi: 10.3389/fsurg.2025.1718589 (PMC12819706; doi:10.3389/fsurg.2025.1718589)
Supplement: Supplementary file 2 [file Datasheet2.zip › Supplementary Data 2/MR_pipeline_p5e-8/forest_plots/ieu-b-111.finngen_R12_L12_PILONIDALCYST.pdf]

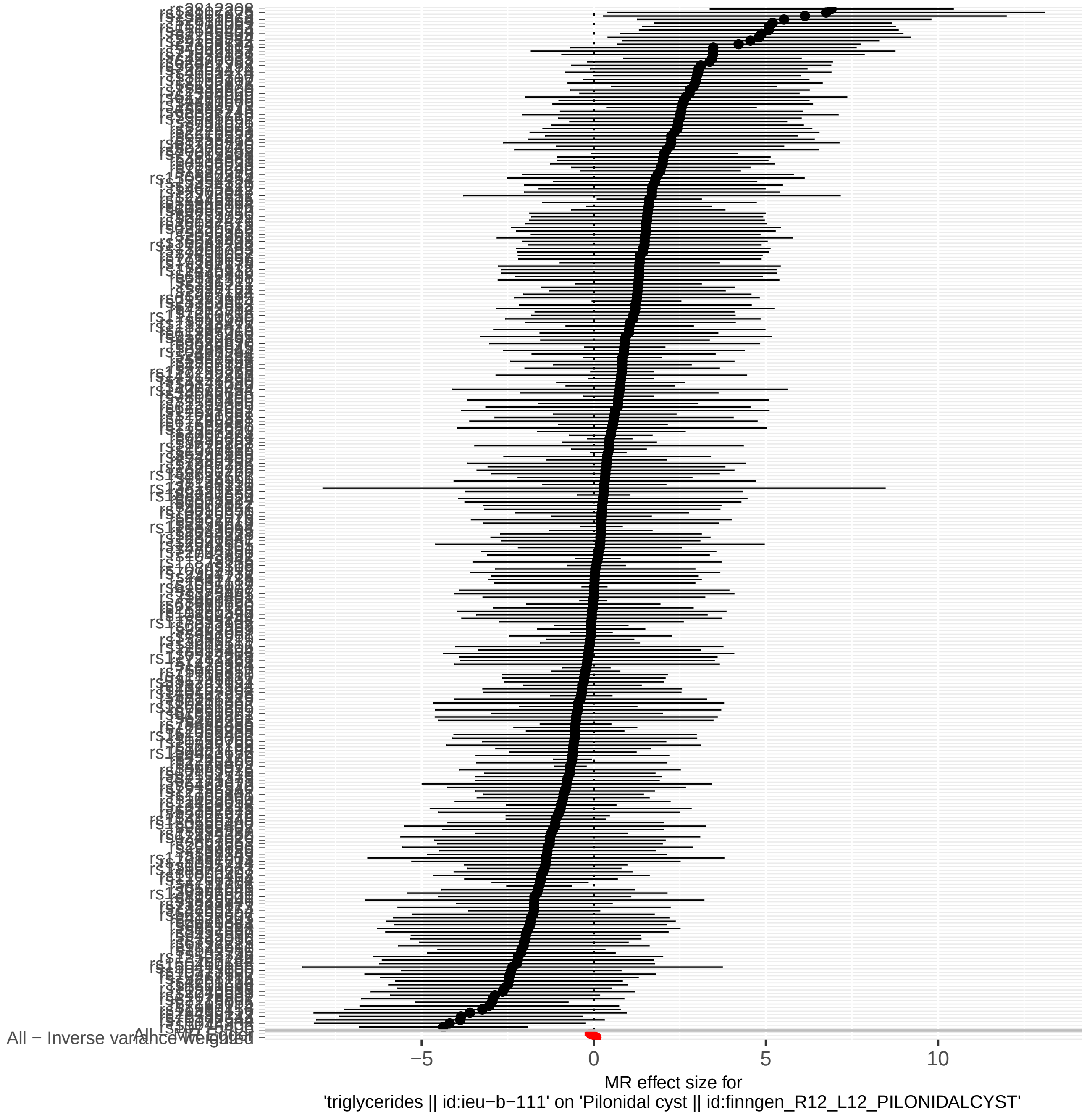
All − Inverse variance weighted
−5
0
5
10
MR effect size for
'triglycerides || id:ieu−b−111' on 'Pilonidal cyst || id:finngen_R12_L12_PILONIDALCYST'
